# Bidirectional two-sample Mendelian randomization study of causality between rheumatoid arthritis and myocardial infarction

**DOI:** 10.3389/fimmu.2022.1017444

**Published:** 2022-12-02

**Authors:** Hao-Yang Guo, Wei Wang, Hui Peng, Hui Yuan

**Affiliations:** ^1^ School of Public Health, Wannan Medical College, Wuhu, Anhui, China; ^2^ Department of Science and Technology, The First Affiliated Hospital of Wannan Medical College (Yijishan Hospital of Wannan Medical College), Wuhu, Anhui, China

**Keywords:** rheumatoid arthritis, myocardial infarction, bidirectional, two-sample Mendelian randomization study, causal association

## Abstract

**Background:**

Epidemiological evidence suggests an association between rheumatoid arthritis (RA) and myocardial infarction (MI). However, causality remains uncertain. Therefore, this study aimed to explore the causal association between RA and MI.

**Methods:**

Using publicly available genome-wide association study summary datasets, bidirectional two-sample Mendelian randomization (TSMR) was performed using inverse-variance weighted (IVW), weighted median, MR-Egger regression, simple mode, and weighted mode methods.

**Results:**

The MR results for the causal effect of RA on MI (IVW, odds ratio [OR] = 1.041, 95% confidence interval [CI]: 1.007–1.076, *P* = 0.017; weighted median, OR = 1.027, 95% CI: 1.006–1.049, *P* = 0.012) supported a causal association between genetic susceptibility to RA and an increased risk of MI. MR results for the causal effect of MI on RA (IVW, OR = 1.012, 95% CI: 0.807–1.268, *P* = 0.921; weighted median, OR = 1.069, 95% CI: 0.855–1.338, *P* = 0.556) indicated that there was no causal association between genetic susceptibility to MI and an increased risk of RA.

**Conclusion:**

Bidirectional TSMR analysis supports a causal association between genetic susceptibility to RA and an increased risk of MI but does not support a causal association between genetic susceptibility to MI and an increased risk of RA.

## 1 Introduction

Rheumatoid arthritis (RA) is a chronic systemic autoimmune disease that affects several tissues and organs and causes chronic synovial inflammation, eventually leading to joint destruction, chronic disability, and reduced life expectancy (1). RA results from the interaction of genetic susceptibility, environmental factors, and immune factors, among which genetic factors determine 50–60% of the risk of RA (2, 3). Myocardial infarction (MI) is a cardiovascular disease in which the formation of plaques on the inner walls of the arteries leads to a decrease in blood flow to the heart, and long-term ischemia and hypoxia result in the death of myocardial cells (4, 5). Some observational studies have shown a close relationship between RA and MI. A cross-sectional study by Dougados et al. (6) of 4,586 RA patients enrolled in 17 countries showed a high prevalence of comorbidities among RA patients, with 6% (95% confidence interval [CI] 5.3%–6.8%) having a history of MI or stroke. In a 10-year cohort study, Lindhardsen et al. (7) found that patients with RA had an increased risk of MI of approximately 70% compared to the general population after adjusting for factors such as sex, age, and socioeconomic status. Among post-MI patients, those with RA have a poor prognosis and an increased risk of death, which is positively correlated with RA duration and steroid dosage (8). A systematic review and meta-analysis showed (9) that the risk of MI increased significantly in patients with RA (relative risk: 1.69, 95% CI 1.50–1.90). However, a cohort study by Rostami et al. (10) found that the weighted genetic risk score of RA had contributed little to the morbidity risk of MI.

Causal inferences from observational studies are susceptible to bias owing to reverse causality and potential confounders (11), which weakens our understanding of the causal association between RA and MI. Randomized controlled trials (RCTs) are the gold standard for causal inferences in epidemiological studies. Some RCTs are difficult to perform owing to medical ethics, subject selection, and extrapolation of results. Mendelian randomization (MR) is a technique that uses genetic variation as an instrumental variable (IV) to assess whether an observational association between exposure factors and outcomes is consistent with a causal effect (12). Genetic variation is not affected by the external environment, social behavior, or other factors, and it is a long-term and stable exposure factor. MR can avoid the effect of confounding factors and reverse causal association on the correlation effects in observational studies, and minimize bias. Published data were collected in this study and bidirectional TSMR analysis was used to determine whether there was a bidirectional causal association between RA and MI.

## 2 Materials and methods

### 2.1 Data sources

Relevant genome-wide association study (GWAS) datasets were obtained from the IEU OpenGWAS project (https://gwas.mrcieu.ac.uk). The GWAS dataset for RA was derived from GWAS analysis and included 13,838 cases and 33,742 controls of European ancestry (13). The GWAS dataset for MI was derived from another GWAS analysis and included 14,825 cases and 44,000 controls of European ancestry (14) (**Supplementary Table 1**).

### 2.2 Screening of IVs

Single nucleotide polymorphisms (SNPs) were used as IVs. P-value (*P*<5.0×10^-8^) was set. To avoid linkage disequilibrium (LD) bias, LD with significant SNPs associated with exposure factors must meet the following conditions: r^2^<0.001, and genetic distance of 10000 kb. Significant SNPs associated with exposure factors were extracted in the GWAS dataset of outcome variables, and the resulting IVs were recorded with information on the effect allele, allele effect sizes (beta), standard error, and p-value. The F-statistic was used to test the strength of each IV and was calculated using the following formula: F = R^2^(N−2)/(1−R^2^), where R^2^ is the proportion of the exposure factor variation explained by each IV, and N is the sample size of the expourse dataset (15). When F>10, there is no weak IVs bias (16).

### 2.3 Research design

To better estimate the causal effect, three key assumptions should be met when SNPs are used as IVs in the TSMR analysis (17) (1): IVs must be closely related to exposure factors; (2) IVs are independent of confounding factors; and (3) IVs can only influence the outcome through exposure and not through other pathways.

### 2.4 Statistical analysis

Summary statistics for the exposure and outcome datasets were harmonized such that the effect of SNPs on exposure and the effect of SNPs on outcome corresponded to the same alleles. TSMR analyses using inverse-variance weighted (IVW), weighted median, MR-Egger regression, simple mode, and weighted mode methods were performed to infer causal associations. We used the IVW as the primary method for MR. When each genetic variation met the IV hypothesis, the IVW method combined the Wald ratio estimates of the causal effects of different SNPs and provided a consistent estimate of the causal effect of exposure on the outcome (18). The results of the IVW method were most reliable when there was no horizontal pleiotropy of the IVs (19). When at least half of the SNPs are effective IVs, the weighted median can provide a consistent estimate of the causal effect (20). MR-Egger regression is used to confirm whether horizontal pleiotropy of IVs exists, and its intercept represents the effect estimate of horizontal pleiotropy (21). When the IVs have horizontal pleiotropy, the MR-Egger regression can still obtain an unbiased estimation of causal association. The weighted median method improves the accuracy of the results compared to the MR-Egger method (22). Simple mode and weighted mode were performed as complementary analyses (23). The Mendelian randomization pleiotropy residual sum and outlier (MR-PRESSO) test was used to detect and correct horizontal pleiotropy by removing the outliers (24). MR power analysis was performed using an online tool (http://cnsgenomics.com/shiny/mRnd/) (25). Statistical analysis was performed using R (version 4.1.0) and R packages (TwoSampleMR and MR-PRESSO). The test level α was 0.05 (*P* < 0.05), and the difference was statistically significant.

## 3 Results

### 3.1 Causal effects of RA on MI

#### 3.1.1 SNPs: Basic information

**Figure 1:**
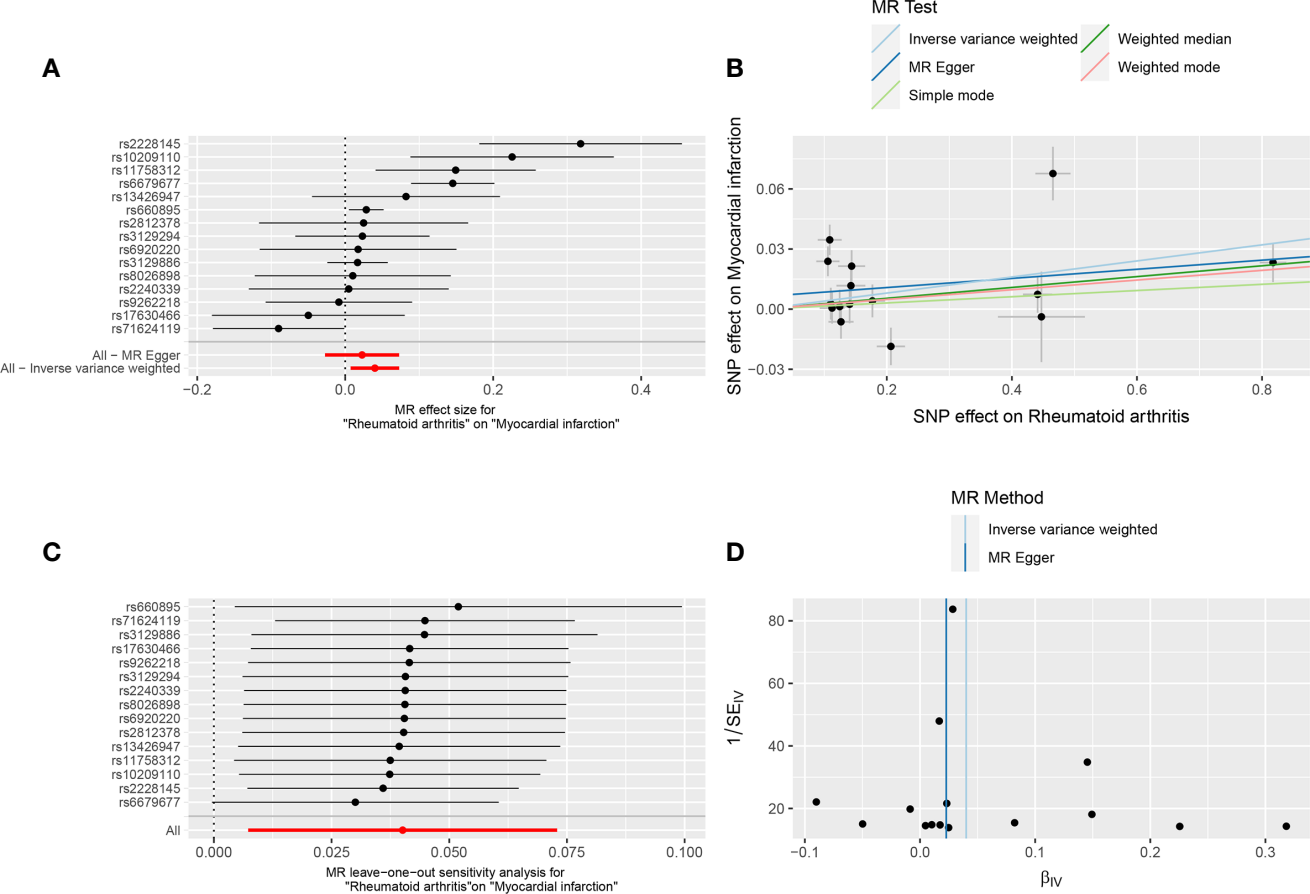
Forest plot **(A)**, scatter plot **(B)**, sensitivity analysis **(C)**, and funnel plot **(D)** of the effect of RA on MI.

RA was the exposure factor, and MI was the outcome variable. In total, 15 SNPs were screened and identified as IVs, with F values greater than 10. The variance explained by these IVs was 44% for RA (**Supplementary Table 2**). The intercept of the MR-Egger regression can be used as an indicator to test whether horizontal pleiotropy of the IVs influences the results of TSMR analysis. The intercept was close to 0 (Egger intercept = 0.006, *P* = 0.384) (**Table 1**), indicating that there was no horizontal pleiotropy of the IVs, and it was unlikely to influence the results of the TSMR analysis (**Figure 1B**).

**Table 1:** Heterogeneity test and Horizontal pleiotropy test.

Exposure	Outcome	Heterogeneity test (MR-Egger)			Heterogeneity test (IVW)			Horizontal pleiotropy test (MR-Egger)	
		Cochran’s Q	Q_df	*P*	Cochran’s Q	Q_df	*P*	Intercept	*P*
RA	MI	51.36	13	1.74E-06	54.57	14	1.03E-06	0.006	0.384
MI	RA	14.62	7	0.041	15.18	8	0.056	-0.01	0.621

#### 3.1.2 Two-sample Mendelian randomization analysis

The MR results supported a causal association between genetic susceptibility to RA and an increased risk of MI. The MR analysis had 81% statistical power. In the absence of horizontal pleiotropy of IVs, IVW was used as the primary method to estimate the causal association between genetic susceptibility to RA and an increased risk of MI (IVW result: OR = 1.041, 95% CI: 1.007–1.076, *P* = 0.017). Results of the other methods included: MR-Egger, OR = 1.023, 95% CI: 0.973–1.076, *P* = 0.389; weighted median, OR = 1.027, 95% CI: 1.006–1.049, *P* = 0.012; simple mode, OR = 1.016, 95% CI: 0.968–1.065, *P* = 0.534; and weighted mode, OR = 1.024, 95% CI: 1.003–1.046, *P* = 0.042 (**Table 2**, **Figures 1A, B**).

**Table 2:** Mendelian randomization analysis of causal association between RA and the risk of MI.

Methods	SNPs	Beta	SE	OR (95%CI)	*P*
MR-Egger	15	0.023	0.026	1.023 (0.973,1.076)	0.389
weighted median	15	0.027	0.011	1.027 (1.006,1.049)	0.012
IVW	15	0.040	0.017	1.041 (1.007,1.076)	0.017
Simple mode	15	0.015	0.024	1.016 (0.968,1.065)	0.534
Weighted mode	15	0.024	0.011	1.024 (1.003,1.046)	0.042

#### 3.1.3 Heterogeneity test and sensitivity analysis

IVW and MR-Egger regression analyses were used to detect heterogeneity between IVs. Heterogeneity was quantified using Cochran’s Q test. *P* < 0.05 indicated significant heterogeneity. If there was heterogeneity between the IVs, the random-effects IVW model was used to estimate causal effects (26). MR-Egger regression (Cochran’s Q = 51.36, *P* = 1.74E-06) and IVW (Cochran’s Q = 54.57, *P* = 1.03E-06) (**Table 1**, **Figure 1D**) indicated that there was heterogeneity between the IVs, and the random-effects IVW model was used to estimate the causal effect (*P* = 0.017). The MR-PRESSO test was used to remove the outlier SNPs (rs2228145, rs6679677, and rs71624119) and to estimate the causal effect of TSMR after correction for outliers (*P* =0.019) (**Supplementary Table 3**).

The sensitivity analysis used the leave-one-out method to remove SNPs one by one, and the causal effects of the remaining SNPs were compared with the TSMR analysis results of all SNPs to determine whether the causal association was due to a single IV, indicating that the TSMR analysis results were robust (**Figure 1C**).

### 3.2 Reverse TSMR analysis

**Figure 2:**
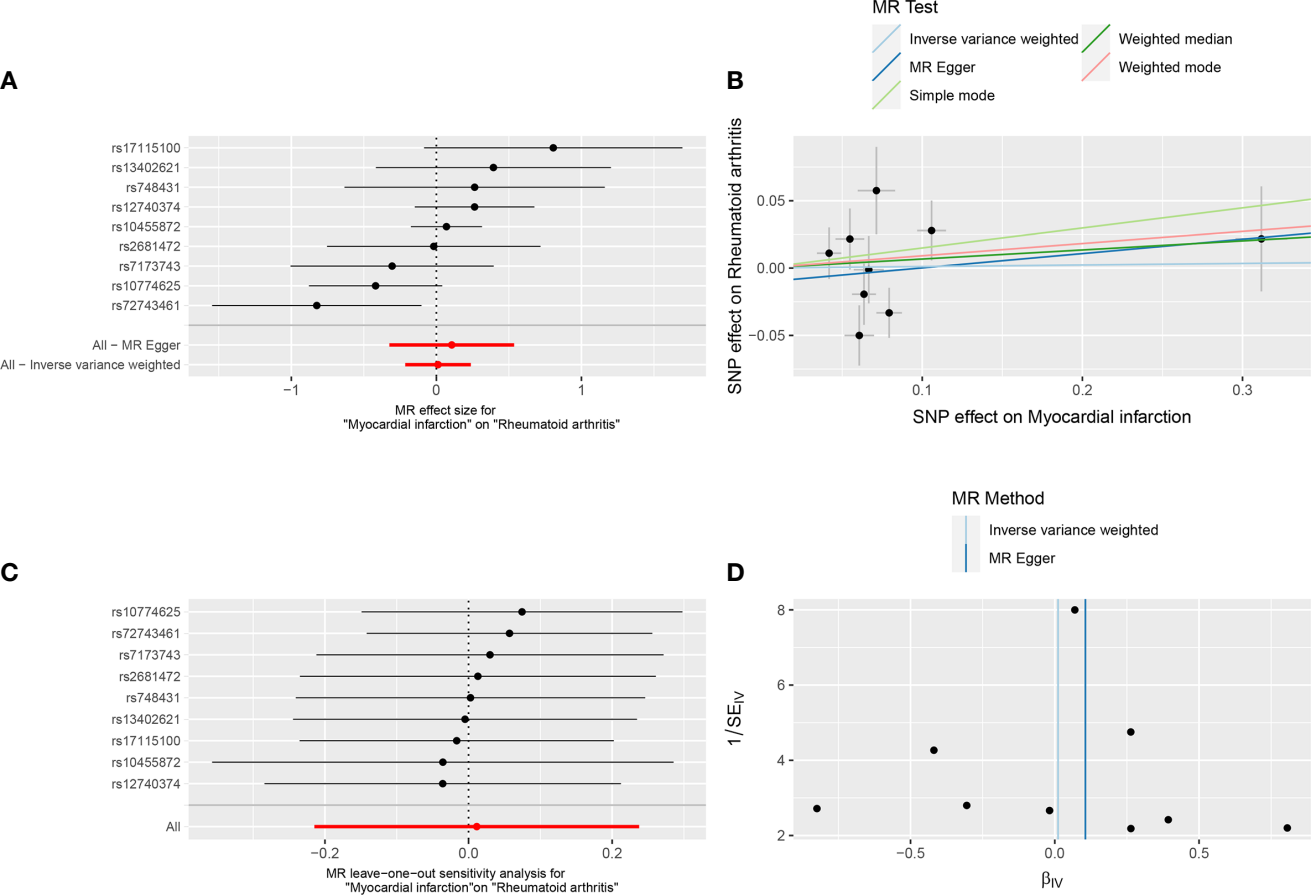
Forest plot **(A)**, scatter plot **(B)**, sensitivity analysis **(C)**, and funnel plot **(D)** of the effect of MI on RA.

In reverse TSMR, MI was the exposure factor, and RA was the outcome variable. In total, 9 SNPs were screened and identified as IVs, with F values greater than 10. The variance explained by these IVs was 2.6% for MI (**Supplementary Table 4**). The horizontal pleiotropy test (Egger intercept = −0.01, *P* = 0.621) (**Table 1**) indicated that there was no horizontal pleiotropy for the IVs. MR results did not support a causal association between genetic susceptibility to MI and an increased risk of RA (IVW, OR = 1.012, 95% CI: 0.807 – 1.268, *P* = 0.921). Results of other methods included: MR-Egger, OR = 1.112, 95% CI: 0.723–1.710, *P* = 0.643; weighted median, OR = 1.069, 95% CI: 0.855–1.338, *P* = 0.556; simple mode, OR = 1.161, 95% CI: 0.763–1.765, *P* = 0.505; and weighted mode, OR = 1.095, 95% CI: 0.871–1.377, *P* = 0.458 (**Table 3**, **Figures 2A, B**). Among the heterogeneity test results, MR-Egger regression showed relatively small heterogeneity (Cochran’s Q =14.62, *P* = 0.041), whereas IVW (Cochran’s Q = 15.18, *P* = 0.056) did not find heterogeneity between IVs (**Table 1**, **Figure 2D**). MR-PRESSO indicated that there was no horizontal pleiotropy for the global test (RSS_obs_ = 18.31, *P* = 0.115) (**Supplementary Table 3**), and no outliers were observed. The leave-one-out method was used for sensitivity analysis, and the results of the TSMR analysis were reliable (**Figure 2C**).

**Table 3:** Mendelian randomization analysis of causal association between MI and the risk of RA.

Methods	SNPs	Beta	SE	OR (95%CI)	*P*
MR-Egger	9	0.106	0.219	1.112 (0.723,1.710)	0.643
weighted median	9	0.067	0.114	1.069 (0.855,1.338)	0.556
IVW	9	0.011	0.115	1.012 (0.807,1.268)	0.921
Simple mode	9	0.149	0.214	1.161 (0.763,1.765)	0.505
Weighted mode	9	0.091	0.117	1.095 (0.871,1.377)	0.458

## 4 Discussion

This study used the bidirectional TSMR method to analyze published GWAS datasets and determine whether a bidirectional causal association exists between RA and MI in the European population. Our results supported a causal association between genetic susceptibility to RA and an increased risk of MI (IVW, OR = 1.041, 95% CI: 1.007 – 1.076, *P* = 0.017). However, our results did not support a causal association between genetic susceptibility to MI and an increased risk of RA (IVW, OR =1.012, 95% CI: 0.807 – 1.268, *P* = 0.921). In the sensitivity analysis, the MR results were robust and reliable.

Several possible reasons have been proposed to explain the association between RA and MI in observational studies. First, MI in RA may be associated with RA-related inflammatory reactions (27). Inflammation can promote the development of atherosclerotic plaques, increase vulnerability, and promote thrombosis (28). Acute phase reactants in patients with RA cause synovitis, and elevated levels of inflammatory cytokines such as tumor necrosis factor α (TNF-α) and interleukin 6 (IL-6) induce atherosclerotic changes and endothelial function damage, leading to an increased risk of MI (29). The TNF-α-induced signaling pathway plays an important role in cellular responses to inflammation and injury, leading to vascular dysfunction and adverse reactions to cardiac remodeling after MI (30). A case-control study showed (31) that the plasma concentrations of IL-6 and IL-6 binary complexes could predict the risk of MI. Early active RA treatment can better control the inflammation. Glucocorticoids (GC) are commonly used as immunosuppressive agents for the treatment of RA. Their anti-inflammatory and immunosuppressive effects can reduce the damage to blood vessels caused by inflammation. However, the long-term use of GC leads to a series of adverse reactions, including cardiovascular diseases (32, 33). A case-control study by Wilson et al. (34) showed that an increase in the cumulative and average daily GC doses was associated with an increased risk of MI. A cohort study by Pujase-Rodriguez et al. (33) showed that even at a lower dose of GC (< 5 mg), the risk of MI increased. Second, there is an increased risk of cardiovascular risk factors (hypertension, dyslipidemia, and diabetes) in RA patients, which, together with inflammation, can promote the formation of atherosclerosis in RA patients and may further contribute to the development of MI (35). Third, genetic factors may also play a role in the association between RA and MI. Vascular endothelial growth factor, a promoter of normal and abnormal angiogenesis, plays an important role in RA pathogenesis (36). Studies by Chen et al. (37) have shown that SNPs in the vascular endothelial growth factor A promoter regions (-2578 and -460) are associated with an increased risk of MI in patients with RA. Palomino-Morales et al. (38) found that the IL6 -174 gene polymorphism is associated with subclinical atherosclerosis, and RA patients with the IL6 -174GG genotype have severe endothelial dysfunction. Methylene tetrahydrofolate reductase 1298 A>C gene polymorphism increases the risk of atherosclerosis in patients with RA (39). Our MR results did not reveal a causal association between genetic susceptibility to MI and an increased risk of RA, and more studies should be conducted in the future.

MR uses genetic variation to estimate the health consequences of the phenotypes affected by these genetic variations (40). This is a relatively novel epidemiological approach that uses genetic variation to infer the causal association between exposure factors and outcome variables. MR provides a way to investigate associations without the typical biases inherent in observational epidemiological studies, such as reverse causal association and potential confounders. Our results differ from the MR results of Fokina et al. (41), probably because of the different GWAS datasets selected, and newer, larger GWAS studies will be necessary in the future.

This study had some limitations. First, the MR results were based on the European population, and extrapolation of the results is limited. Whether a causal relationship exists in other populations needs to be confirmed by further research. Second, SNPs used for analysis may be correlated with other traits due to genetic polymorphisms and generate confounding bias, which may affect causal inference. Third, the strength of the IV depends on the sample size of the GWAS, and a larger scale GWAS is required to determine more genetic variation for MR.

## 5 Conclusion

In summary, bidirectional TSMR analysis supports a causal association between genetic susceptibility to RA and an increased risk of MI, but does not support a causal association between genetic susceptibility to MI and an increased risk of RA. However, due to the limitations of the study, further research is necessary.

## Data availability statement

The original contributions presented in the study are included in the article/**Supplementary Material**. Further inquiries can be directed to the corresponding author.

## Author contributions

H-YG: conceptualization, investigation, data curation, writing - original draft. WW: conceptualization, investigation, writing - original draft. HP: investigation, writing - review and editing. HY: conceptualization, writing - review and editing. All authors contributed to the article and approved the submitted version.

## Funding

This work was supported by grants from Anhui Provincial Natural Science Foundation (No. 1808085QH251) and Horizontal scientific research project of Wannan Medical College (No. H2020001).

## Acknowledgments

We thank all participants for the study we conducted.

## Conflict of interest

The authors declare that the research was conducted in the absence of any commercial or financial relationships that could be construed as a potential conflict of interest.

## Publisher’s note

All claims expressed in this article are solely those of the authors and do not necessarily represent those of their affiliated organizations, or those of the publisher, the editors and the reviewers. Any product that may be evaluated in this article, or claim that may be made by its manufacturer, is not guaranteed or endorsed by the publisher.
